# The Varioloid Disease

**Published:** 1838-10-10

**Authors:** N. Chapman

**Affiliations:** Professor of the Theory and Practice of Physic, in the University of Pennsylvania


### THE MEDICAL EXAMINER.

DEVOTED TO MEDICINE, SURGERY, AND THE COLLATERAL SCIENCES.

EDITED BY J. B. BIDDLE, M.D., M. CLYMER, M.D., AND W. W. GERHARD, M.D.

No. 21.] PHILADELPHIA, WEDNESDAY, OCTOBER 10, 1838. [Vol. I

### THE VARIOLOID DISEASE.

*By* N. Chapman, M. D., *Professor of the Theory and Practice of Physic, in the University of Pennsylvania.*

(Concluded from page 315.)

Notwithstanding all which has been said, in contemplating the subject in its several relations, I can discern no just grounds, at least in the present uncertainty of it, for abandoning vaccination, and still less for reverting to the process of variolation. Excepting in the degree of protective power, which seems to be much in favour of inoculation, though in this respect the results vary considerably in some places, the superiority in every other view is indisputably with vaccination. Exclusively of diverse considerations by which it is recommended, too obvious to be noticed here, it is a process mild in its general character, rarely inducing unpleasant consequences, and never proves fatal. But of inoculated small-pox, we are told by Willan, one in two hundred and fifty dies, and several distinguished English writers have made it as one in a hundred.* Of natural small-pox, one in four or five ordinarily, and when epidemic, one in two or three is lost on an average, according to the best computation. Distinct from its mortality, it may be objected to variolation, that it occasionally entails the most lamentable effects, developing scrofula and other loathsome diseases,—causes the loss of sight, and is destructive, by its disfiguration of personal comeliness.

By variolation, supposing the disease in this form to be infectious, the sources of contagion are, moreover, multiplied, each case proving a new point from which the disease may emanate, so that, though individuals were benefitted by the mitigating influence of the process, the aggregate of mischief was actually increased. Calculations made by Heberden, without any reference to this question, show, that subsequently to the introduction of inoculation, *ninety-five* persons died of small-pox, in London, out of every thousand; whereas, the average number, antecedently to it, was only seventy in the thousand. Corroborative of this, it is shown, that in Spain, where the practice of inoculation was scarcely ever admitted, small-pox has caused less mortality in proportion to the population, than in any country in Europe.

Even in this state of amelioration, the sort of evil small-pox proved, may be determined, when it is told, that it destroyed nearly fifty thousand individuals annually in the kingdom of Great Britain only, occasioning every fourth death on the bills of mortality. Lettsome computed the mortality in Europe at two hundred and ten thousand; and Bernouilli, an Italian authority, throughout the world at six hundred thousand annually. By vaccination, though limited by vulgar prejudices, this frightful expenditure of human life has been greatly abridged. What is the extent of its whole effects in this respect, I have no means of determining. But we have some striking facts in relation to particular countries, where vaccination was enforced by law. Thus it appears from official reports, that in Copenhagen the mortality had been reduced from five thousand five hundred in twelve years, to about one hundred and fifty-eight in sixteen years, and finally small-pox became extinct in Denmark. The same happened in the principality of Anspach. In Prussia, the number of deaths was diminished from forty thousand, to three thousand annually; and in Bavaria, only five persons died of the disease in eleven years.

An extension of the plan by which small-pox was eradicated in these countries, it has been thought, would as certainly do it as regards the whole world, and by a wise, and cordial co-operation in this mighty work of benevolence, this terrible scourge of humanity, might in a few years, be so completely annihilated, as to leave behind only its name, and the story of its former ravages. But these hopes are not well founded. Not to urge the difficulty of enacting and enforcing penal provisions against inoculation, or other modes of introducing or continuing the disease, under free governments, there is an insuperable obstacle to its extinguishment in the circumstance which has not been adverted to, that it occasionally arises, as it were, de novo, in an epidemic shape, which no regulations can prevent or even repress. This very city, admitting the late epidemic to have been small-pox, affords an illustration of the force of this objection to the scheme. As I have more than once said, for a long period, it escaped entirely the disease by a common agreement of the physicians not to variolate, aided in the design by strict quarantine, and some other regulations, to exclude it. Yet the epidemic burst out among us, and rendered, at once, nugatory all our well-meant efforts. The same has happened in those countries of Europe, which, by similar means, enjoyed for a time, a like immunity. Nevertheless, what cannot be altogether prevented, may be mitigated, and so far it is our duty to exercise our influence, and especially by discouraging the practice of inoculation.

Every other objection aside to variolation, I should be exceedingly distrustful in the present state of our knowledge of the subject, of the genuineness of the virus now to be procured. No one denies that of the precise nature of the epidemic, some doubts may reasonably be entertained, and who can foresee whether it shall prove a security

* These statements, I presume, have reference to the results among the out-door poor of England, with whom no advantages can be commanded. Certainly much greater success has been attained in the small-pox hospitals of that and other countries, and in private practice among persons in comfortable circumstances; where proper skill and attention were exercised, the fatality of the operation had become exceedingly inconsiderable.

against real small-pox? As to vaccination, we have the best evidence of its virtue, so far, at least, as regards the preservation of life; and it seems to me that common prudence requires, that it should not be exchanged, till all uncertainty is removed.

It has been strongly affirmed, that there is no evidence to warrant the popular notion, that vaccination is the parent of certain foul eruptions, or that in the progress of time, the susceptibility to variola may be revived in the system, or the activity of the virus weakened. Experience, it is alleged, so far as it extends, confutes each of these impressions; and that, like the rest of the objections to vaccination, they are, with the qualifications already stated, gratuitous and unfounded. The attention of Willan to the first of these points, and his ample opportunity of deciding it, are well known. By him we are expressly informed, that he is not sensible that any new affections of this kind have been generated since the prevalence of vaccination, or that the old disorders had become more numerous or virulent. But, directly the reverse, as to the latter at least, the distemperatures of the skin have diminished. It is also a remarkable fact, which he cites to the same purport, that, in Gloucestershire, where the vaccine affection has existed longer than elsewhere, no such complaints are heard of.

As to the second point it is averred, that, inquiries very extensively carried on in Britain, prove conclusively, that time exercises no influence whatever over the process, the instances of failure not being more in the old, than recent vaccinations. Treating of this question, Thompson says:—"Nothing has occurred, so far as I have been able to perceive or learn, to warrant the supposition, that the modifying or preventive powers of vaccination are weakened or exhausted by time: on the contrary, the present epidemic has been observed to attack those chiefly who were under ten years of age." In refutation of the third allegation he asserts as a fact, that the virus used at the Royal Dispensary at Edinburgh for eighteen years, was still as efficient as when first collected, though it had passed, during this period, through a succession of at least nine hundred individuals. Marshall, whose work was published some years ago, declares the same in relation to the virus employed in London, which, taken from a cow in 1799, has ever since been kept up, and been probably transmitted through some hundreds of thousands of systems. It appears, indeed, that this is the source of most of the vaccine matter now in the world. During the same year, four thousand cases were propagated from it, and these furnished a stock to supply the whole British empire, including the army and navy, and also most other countries whither it was sent.

In despite of what has been said on these several points, and sustained as each is, by striking facts and high authorities, I am not myself entirely satisfied that some delusion or fallacy does not exist in relation to them. Gregory has very recently stated, that for about five years after a successful vaccination, the system appears to be wholly insensible to a repetition of the process. But at the expiration of ten years, the skin becomes irritated on an application of the virus, followed in a few days by a pointed or acuminated vesicle with an areola of irregular figure, the whole prematurely perishing. The system here suffers for a time, on some occasions, much constitutional disturbance, attended by swellings of the axillary glands. Cases, however, do occur under these circumstances, where the revaccination runs a perfectly regular course, both as the local and general affection, and matter is furnished by the vesicle, capable of propagating the genuine disease. To these facts others may be added of equal or greater force. The frequent occurrence of small-pox after vaccination in the armies of the German States, has recently induced several of the governments to direct a general revaccination of the troops. As regards those of Wurtemburg, of which I have only received any account, it is stated by Professor Hein, that in sixteen hundred and eighty-three individuals, in whom the operation was repeated, in thirty-four in each hundred it completely succeeded—in twenty-two with modified results, and in forty-four it utterly failed. Of five hundred and seventy-seven who were revaccinated, with entire success, two hundred and ninety-three had perfect cicatrices—in one hundred and sixteen, imperfect—and in one hundred and sixty-eight, there were no scars at all. Three hundred and thirty-six, revaccinated with unsatisfactory results, one hundred and ninety-three had good marks, one hundred and thirty-four defective traces only, and thirty-nine no vestige of the kind. Finally, of seven hundred and forty of the revaccinated, without any effect, three hundred and eighty-two showed good, two hundred and twenty-five imperfect, and one hundred and thirty-six no cicatrices.

Now, from these facts it is probable, perhaps certain, that, by time, the system regains, in many instances at least, its sensibility to the vaccine infection, and it may be presumed, in the same way, to that of small-pox. The lesson is hence inculcated, to test it in all cases by revaccination.

It also has of late been questioned, whether the virus now in use, has not become effete or less active, and a resort for a supply to the original source recommended. But the cow seems at present to be exempt from the disease, or at least the most extensive researches in Europe could not detect it in that animal within the last year.

Embarrassed as it is by difficulties, still on the whole, as the result of the best examination which I am capable of giving to the subject, I cannot help urging confidence in vaccination as adequate to many of the purposes originally professed. Let me be distinctly understood. My meaning is, as affording protection against genuine small-pox, or that form of the disease which existed antecedently to this new and anomalous epidemic, and though it does not entirely control that, it so mitigates its force, as to preserve life. Time had rather accumulated and confirmed, than impaired the evidence of its validity, and such I am authorized to state was the opinion of Jenner himself, supported by the official reports of the Board of the National Vaccine Establishment of London, and of the Commission of Paris. Be it indeed admitted, that vaccination is an imposition, all experimental inquiry is futile, our senses are illusive, medical testimony a fallacy, and we must surrender ourselves up to incredulity and scepticism, vacant and unprofitable. But such is not the case, and our prospects of the future are most encouraging.

The present epidemic, whatever may be its nature, is passing away, and I trust we shall have only to contend against small-pox, in its older and ordinary forms. This happening, and vaccination being duly attended to on our parts, I apprehend, that all difficulties may be overcome, all cavils silenced, and all prejudices removed on the subject. By adverting to the loose and indiscriminate manner in which vaccination has been practised, by the heads of families, by the clergy, by old women, and other benevolent, though very incompetent, personages, we ought rather to be surprised that the number of failures have been so small, in a process which, from its delicacy, requires to be superintended by all the powers of discrimination and skill. Every good is interwoven with some portion of evil. The singular mildness of vaccination, by taking it out of professional hands, has undoubtedly very much detracted from its utility, and exposed it to some very unjust imputations. But, a reformation may be instituted in this respect. Considering how much our pride is interested in a discovery, which so gloriously illustrates the character of the profession, it is among our first and highest duties to vindicate it against the caprices of fashion, and fluctuations of opinion, till it is firmly established, to be transmitted to posterity, unimpaired, as the noblest monument ever erected by science, to the purposes of human benefit and happiness.

